# Centrally necrotizing breast carcinoma: a rare histological subtype, which was cause of misdiagnosis in an evident clinical local recurrence

**DOI:** 10.1186/1477-7819-10-156

**Published:** 2012-08-01

**Authors:** Fernando Hernanz, Pilar Alonso-Bartolomé, Irene González-Rodilla

**Affiliations:** 1 Department of Surgery, Valdecilla Hospital, Avda Valdecilla s/n, Santander, 39008, Spain; 2 University of Cantabria, Avda, Cardenal Herrera Oria s/n, 39001, Santander, Spain; 3 Department of Radiology, Valdecilla Hospital, Avda Valdecilla s/n, Santander, 39008, Spain; 4 Department of Pathology, Valdecilla Hospital, Avda Valdecilla s/n, Santander, 39008, Spain; 5 Urb Las Pérgolas 10, Sancibrian, Cantabria, 39110, Spain

**Keywords:** Centrally necrotizing carcinoma, Triple-negative breast cancer, Misdiagnosis

## Abstract

Centrally necrotizing carcinoma is a rare subtype of breast carcinoma, which is characterized by an extensive central necrotic zone accounting for at least 70% of the cross-sectional area of the neoplasm. This central necrotic zone, in turn, is surrounded by a narrow rim of proliferative viable tumor cells. We report an unusual clinical situation in which a patient whose evident breast mass suggested an ipsilateral local recurrence and for which numerous attempts to confirm the histological diagnosis had failed. The patient was treated with a radical mastectomy based on clinical suspicion of breast cancer recurrence after an undesirable delay. In this case, the narrow rim of viable malignant tissue had a thickness of 0.5 to 8 mm, and the centrally necrotizing carcinoma had a central zone with a predominance of fibrosis. The special features of this case led to a misdiagnosis and to an evident clinical local recurrence.

## Background

Today, in most patients suffering from breast cancer, treatment is implemented with a histologically confirmed diagnosis and without a diagnostic surgical procedure because a representative sample of the tumor is usually obtained by current radiologic technology. However, in this case, a patient presented with a large breast mass as a clinical form of a breast cancer recurrence and underwent a radical mastectomy based on clinical suspicion of breast cancer recurrence without microscopic confirmation. Subsequently, the mass was diagnosed as a special subtype of invasive ductal carcinoma also termed ‘centrally necrotizing carcinoma’ (CNC) [1]. The special macroscopic characteristics of this tumor subtype could explain why we did not diagnose cancer recurrence despite the numerous attempts that were carried out to obtain some malignant tissue.

## Case presentation

A 53-year-old white woman diagnosed with a triple-negative basal-like invasive ductal carcinoma located at the intersection of upper quadrants of the right breast was treated with breast-conserving surgery having free surgical margins (>1.5 cm) and sentinel lymph node biopsy. Pathologic stage of the tumor was pT1N0M0. Adjuvant chemotherapy and radiotherapy were given.

Seventeen months after the end of the radiotherapy, she presented with a painful mass in her right breast at the surgical bed. There were neither palpable axillary lymph nodes nor elevation of tumor markers. The imaging study with mammography and magnetic resonance imaging (MRI) showed a right breast mass whose pathological study comprising seven breast tissue fragments obtained by vacuum-assisted percutaneous biopsy revealed fat necrosis and fibrosis. These findings were interpreted as sequelae of breast-conserving treatment. Anti-inflammatory treatment was given and close follow-up was recommended.

**Figure 1:**
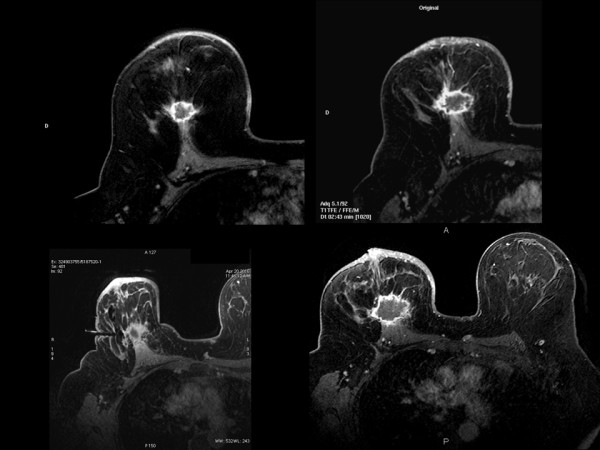
**Series of contrast-enhanced MRI shows a ring enhancement of the mass.** The feature is significantly associated with triple-negative breast cancer. In the lower right corner, MRI-guided vacuum-assisted biopsy that failed to obtain malignant tissue.

**Figure 2:**
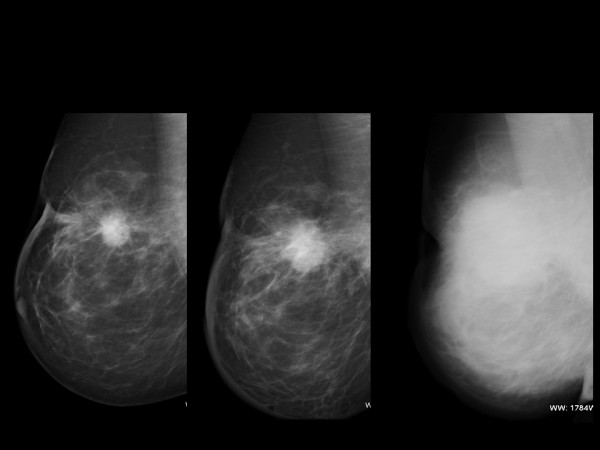
**Sequence of mamographies shows a mass with a rapid growth.** In the last one the whole breast was affected by edema. Skin biopsy did not have any malignant features.

**Figure 3:**
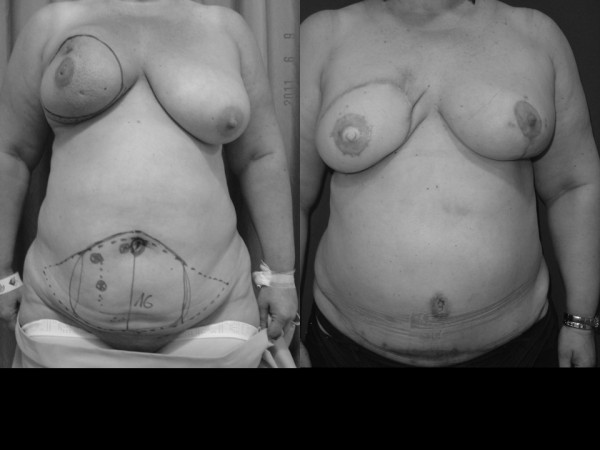
**Appearance of the patient is shown with surgical design before surgery.** Cosmetic result after mastectomy and immediate breast reconstruction with a TRAM unipedicled flap. The left breast was reduced for symmetrization.

After three months, the mass had increased in size and patient complaints had also increased. Complimentary mammography and MRI-guided vacuum-assisted biopsy were performed at the core and at the peripheral enhancement of the mass. Twenty-one breast tissue fragments were obtained and, as with previous biopsies, pathologic diagnosis was confirmed with exclusion of malignancy (Figure 1). Three months later, the clinical situation was worse with edema and slurring, and skin biopsy was performed excluding malignancy again. One year later, another biopsy comprising twenty fragments of nonmalignant breast tissue was carried out. At this time, the patient suffered from an unmanageable constant breast pain, and a new mammogram showed a diffuse affectation of the right breast. The progression of the mass in different mammography images is shown sequentially from left to right in Figure 2. Given these clinical facts, a radical mastectomy was indicated with two possible diagnoses: breast cancer recurrence or radio-induced breast fibrosis. A total mastectomy with resection of the pectoralis major muscle was performed, and the muscle defect and the breast were reconstructed using an ipsilateral unipedicled musculocutaneous TRAM (Transverse Rectus Abdominis Myocutaneous) flap (Figure 3).

### Pathological study

Right breast with a segment of skin weighing 1044 g and measuring 14 × 14 × 7 cm exhibited a retroareolar, well-circumscribed, grayish-white nodular mass with moderate consistency on a cut surface. The mass was 6 cm in size. Microscopically, the tumor was composed of an extensive central acellular hyaline and a necrotic area with some 'ghost’ cells and nuclear debris. This central area occupied more than 70% of the tumor mass and was surrounded by a narrow rim of viable infiltrating ductal carcinoma, poorly differentiated showing lack of tubule lumen formation and high nuclear grade. The thickness of the rim varied from 0.5 to 0.8 cm. Margins were free of malignancy at 1.5 cm minimal distance from the pectoral major muscle.

**Figure 4:**
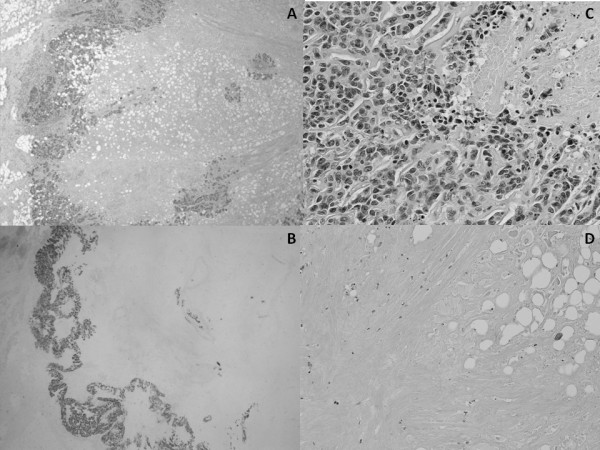
**Microscopic images from pathological study.A.** The tumor shows a unicentric nodule with a prominent, extensive central hipocellular zone, surrounded by a narrow ring of viable tumor cells. **B.** E-cadherin positivity of the malignant cells strongly shows the narrow rim of viable tumor. **C.** Peripheral rim. The tumor cells display high nuclear grade with numerous mitoses. **D.** Central, hypocellular zone with fat necrosis and formation of fibrotic and hyaline material.

**Figure 5:**
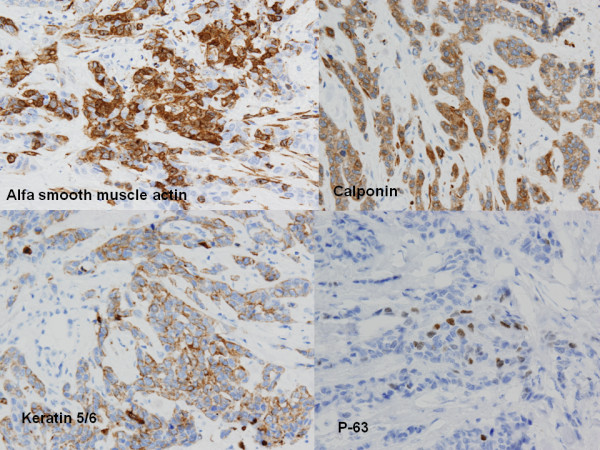
Immunohistochemistry of myoepithelial cells showed α-smooth muscle protein (++/+++), Calponin (++/+++), Keratin 5/6 (+/+++) and P-63 (+/+++).

Immunohistochemical findings showed that estrogen and progesterone receptors and HER2 antibodies failed to stain malignant cells. Proliferative index measured by expression of Ki-67 was positive in 95% of the cells and mutation of suppressor Gen p53 was expressed in 80% (Figure 4). Myoepithelial cell markers showed the expression of P-63 (+/+++), α-smooth muscle protein (α-SMA) (++/+++), Calponin (++/+++), Keratin 5/6 (+/+++), S100 protein positive (+/+++) and glial fibrillary acidic protein (GFAP) negative (Figure 5).

CNC is an uncommon subtype of breast carcinoma which displays the following histological characteristics: it comprises a well-circumscribed unicentric nodule, having an extensive central necrotic zone accounting for at least 70% of the cross-sectional area of the neoplasm; the central zone is surrounded by a narrow rim of proliferative viable tumor cells; and the residual tumor cells show high-grade infiltrating ductal carcinoma, usually accompanied by the component of ductal carcinoma *in situ*. Immunostaining profile can show a myoepithelial immunophenotype characterized by the expression of S100 protein, α-smooth muscle protein (α-SMA) and keratin markers [1-3].

The central necrotic zone can show three histological features: a) predominance of tumor coagulative necrosis with variable degrees of fibrosis and hyaline material, b) predominance of fibrotic or scar-like tissue with a small amount of necrotic tumor debris and c) infarction.

CNC shows highly aggressive biological behavior and rapid clinical progression with very poor prognosis and predilection for metastasizing to lung and brain [1,4,5].

## Conclusions

The CNC special characteristics and, particularly in this case, the central zone pattern with predominance of fibrosis and adiponecrosis, together with the minimal width of the viable tumor rim (0.5 to 0.8 cm) were the cause of the failure to obtain a sample of viable tumor cells in the numerous biopsies that were carried out.

This is the second CNC case published arising in the surgical bed in a patient who had undergone a lumpectomy and radiotherapy. Despite showing all defined clinical and CNC histological characteristics, features of the case contributed to difficulties in diagnosis. The other case was published by Jimenez RE (1) in a series of 34 patients.

## Consent

Written informed consent was obtained from the patient for publication of this report and any accompanying images.

## Abbreviations

α-SMA: α-smooth muscle protein; CNC: Centrally necrotizing carcinoma; GFAP: Glial fibrillary acidic protein; MRI: Magnetic resonance imaging; TRAM: Transverse Rectus Abdominis Myocutaneous.

## Competing interests

The authors declare that they have no competing interests.

## Authors’ contributions

F H carried out the surgical treatment and drafted the manuscript. P A-B revised radiological studies. I G-R carried out the pathological studies. All authors read and approved the final manuscript.
